# Wild Type Beta-2 Microglobulin and DE Loop Mutants Display a Common Fibrillar Architecture

**DOI:** 10.1371/journal.pone.0122449

**Published:** 2015-03-24

**Authors:** Antonino Natalello, Annalisa Relini, Amanda Penco, Levon Halabelian, Martino Bolognesi, Silvia Maria Doglia, Stefano Ricagno

**Affiliations:** 1 Dipartimento di Fisica G. Occhialini and Dipartimento di Biotecnologie e Bioscienze, Università di Milano-Bicocca, P.zza della Scienza 2, Milano, Italy; 2 Dipartimento di Fisica, Università di Genova, via Dodecaneso 33, Genova, Italy; 3 Dipartimento di Bioscienze, Università di Milano, Via Celoria 26, Milano, Italy; 4 CIMAINA e Istituto CNR di Biofisica, Milano, Italy; University of Akron, UNITED STATES

## Abstract

Beta-2 microglobulin (β2m) is the protein responsible for a pathologic condition known as dialysis related amyloidosis. In recent years an important role has been assigned to the peptide loop linking strands D and E (DE loop) in determining β2m stability and amyloid propensity. Several mutants of the DE loop have been studied, showing a good correlation between DE loop geometrical strain, protein stability and aggregation propensity. However, it remains unclear whether the aggregates formed by wild type (wt) β2m and by the DE loop variants are of the same kind, or whether the mutations open new aggregation pathways. In order to address this question, fibrillar samples of wt and mutated β2m variants have been analysed by means of atomic force microscopy and infrared spectroscopy. The data here reported indicate that the DE loop mutants form aggregates with morphology and structural organisation very similar to the wt protein. Therefore, the main effect of β2m DE loop mutations is proposed to stem from the different stabilities of the native fold. Considerations on the structural role of the DE loop in the free monomeric β2m and as part of the Major Histocompatibility Complex are also presented.

## Introduction

Amyloidosis is characterized by the conversion of a protein from its native state into insoluble highly organized fibrillar aggregates, being at the roots of several protein misfolding diseases in man, such as Alzheimer, Parkinson and Huntington diseases [1]. β2-microglobulin (β2m) is the light chain of class I major histocompatibility complex (MHC-I) [2]. It is a 99-residue protein displaying a classic immunoglobulin fold, based on two facing β-sheets that are linked by a disulphide bond. Under physiological conditions, β2m turnover takes place in kidneys, where it is degraded. In case of renal failure, the degradation of β2m does not occur, and the protein accumulates in the blood increasing its concentration up to 50-fold in hemodialysed patients [3]. When such high β2m blood level is retained over the years, the protein self-associates into amyloid fibrils [4], which accumulate around the skeletal joints, bones and muscles. Such condition, known as dialysis related amyloidosis, is characterized by typical symptoms such as movement impairment, bone fragility, and carpal syndrome [4].

Somehow contradictory is the observation that *in vitro* β2m is a very stable protein, reaching mM concentrations without precipitating or aggregating, and yet *in vivo* at much lower concentration β2m turns into amyloid deposits [5]. Although several factors, such as type I collagen and glycosaminoglycans (GAGs) [6–8] have been reported to facilitate β2m aggregation, very little light has been shed on the β2m regions that trigger or mediate the fibril formation.

Many studies have shown the relevance of different β2m stretches in determining the protein aggregation propensity. Among others, the *cis* to *trans* isomerisation of Pro32, in the BC-loop, has been shown to be one of the fundamental steps of the conversion from the native fold to the amyloidogenic intermediate [9]; in keeping with this observation, no *cis*-Pro residues have been observed in the mature β2m fibrils [10]. Moreover, the N-terminal six residues are known to stabilise the β2m fold; accordingly, the natural variant ΔN6 (*i*.*e*. β2m devoid of the first six residues) is highly amyloidogenic [11]. Notably, the natural β2m mutant (D76N), recently discovered, highlights the role of the EF loop in determining β2m stability and aggregation propensity [12].

**Fig 1:**
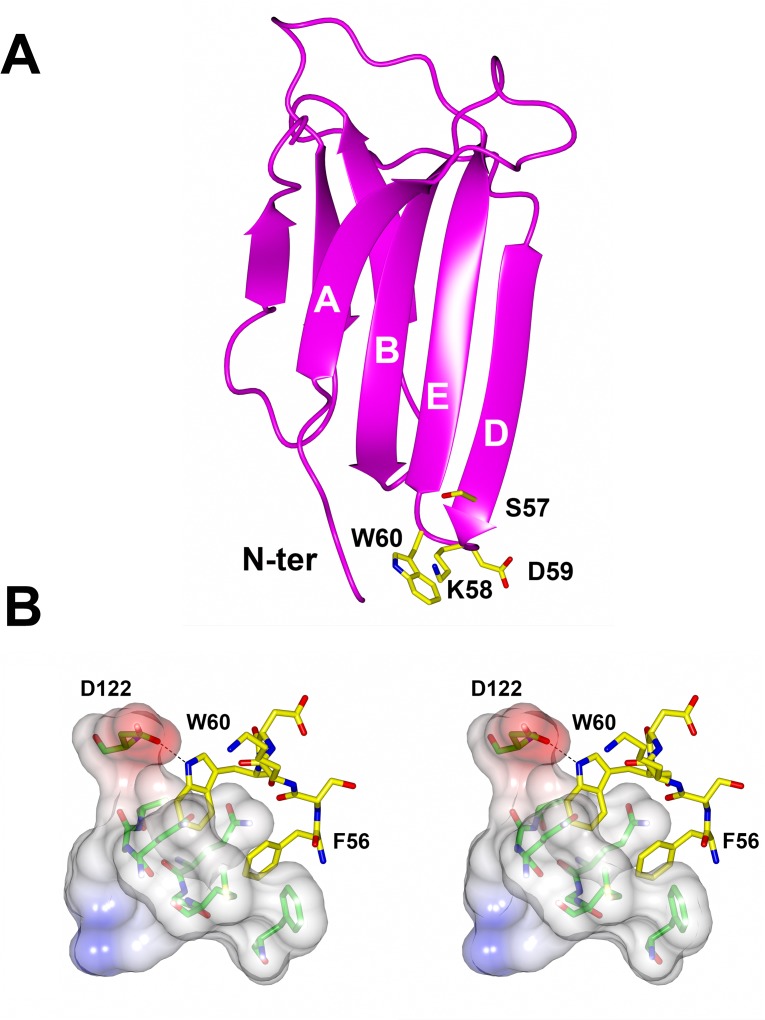
DE loop in monomeric β2m and in interaction within the MHC-I. (A) Ribbon representation of monomeric β2m (PDB code 2YXF). The DE loop residues are shown in yellow sticks. (B) Stereo view of the DE loop and Phe56 (yellow sticks) when interacting with the heavy chain in the MHC-I (electrostatic surface and green sticks). Trp60 is establishing a H-bond with Asp122 from the heavy chain (PDB code 4L29).

Over the years Trp60 and in general the β2m DE-loop (residues 57–60) have been shown to be crucial in determining many of the wild type (wt) β2m biochemical and biophysical properties (Fig. 1A) [13–15]. Trp60 is a highly conserved residue in vertebrate β2m, being primarily involved in the association interface linking β2m to the heavy chain within the MHC-I complex [16]. We recently showed that the interactions within the MHC-I complex greatly stabilise the β2m fold (Fig. 1B) [17]; however, while the DE-loop is designed to play a role in formation of the MHC-I complex, it appears as an element of instability for monomeric β2m in solution. Many data collected over the past few years support such view. First of all, the DE-loop displays a non-ideal backbone geometry [16, 18]. In order to test whether the DE-loop geometry might be relevant for β2m biochemical and aggregation properties, Trp60 was mutated to Gly, to obtain a less strained DE-loop (W60G mutant) [16], and to Val, to verify the effect of the Trp side chain alone (W60V mutant) [18]. Asp59 was mutated to Pro (D59P mutant), to evaluate the effects of an even more rigid DE-loop on the overall β2m molecule [19]. β2m thermodynamic stability, measured by temperature and chemical unfolding, correlates well with the DE-loop geometry [15]. Aggregation kinetics indicate that the lower is the thermodynamic stability, the faster and more abundant is the aggregation [13]. However, other data are indicating some direct role of Trp60 in β2m aggregation: first of all electrospray-mass spectrometry under native conditions showed that while wt β2m in solution presents a non-negligible population of small oligomers, the mutant W60G and W60V showed markedly reduced tendency to native aggregation [15]. Under standard conditions the W60V mutant is aggregating less abundantly than wt β2m [18].

Overall, the above evidence clearly indicates that the DE-loop is a major determinant of β2m fold thermodynamic stability and aggregation propensity. However, one main question about the effect(s) of the DE-loop mutations on β2m amyloid aggregation remains open. The mutations in the DE-loop may result in thermodynamic (de)stabilization of the native protein, which in turn can alter the kinetic barrier(s) along the aggregation process, while the aggregation pathway remains unaltered. An alternative possibility is that such mutations may affect β2m aggregation propensity by opening new aggregation pathways and leading to different and unrelated kind(s) of β2m aggregates. To address this question, we performed a comparative biophysical characterisation of amyloid aggregates formed by wt β2m and DE loop mutants. In particular, the morphology and structural organization of early aggregates and mature fibrils have been analysed by Atomic Force Microscopy (AFM); then, the hydrogen/deuterium (H/D) exchange kinetics and the overall secondary structure content for the fibrils of the four β2m variants have been monitored by Fourier transform infrared (FTIR) spectroscopy. Previous data showed that the DE loop mutations exert a major effect on the stability of the β2m native fold and affect the aggregation kinetics [13, 15] while the experiments here presented indicate that the fibrils of the DE loop variants and of wt β2m have comparable architecture and dynamics, suggesting that mutations at the DE loop effects are limited to the β2m aggregation process, and do not alter the end stage of the aggregation pathway.

## Results

### Amyloid fibril preparation

Purified samples of wt β2m and of the three DE loop variants have been placed under standard aggregation conditions (see methods). Aggregated samples after 24 hours and after one-week incubation have been tested for thioflavin fluorescence (Table 1) and have been analysed by means of AFM and FTIR.

**Table 1:** Amyloid fibril formation of wt β2m, D59P, W60G and W60V β2m variants monitored by ThT fluorescence (given in arbitrary units).

	**wt β2m**	D59P β2m	W60G β2m	W60V β2m
24 hour incubation	40 (± 2)	46 (± 4)	18 (± 1)	29 (± 1)
1 week incubation	79 (± 21)	62 (± 15)	12 (± 2)	69 (± 22)

### Characterisation of the amyloid aggregates by AFM

**Fig 2:**
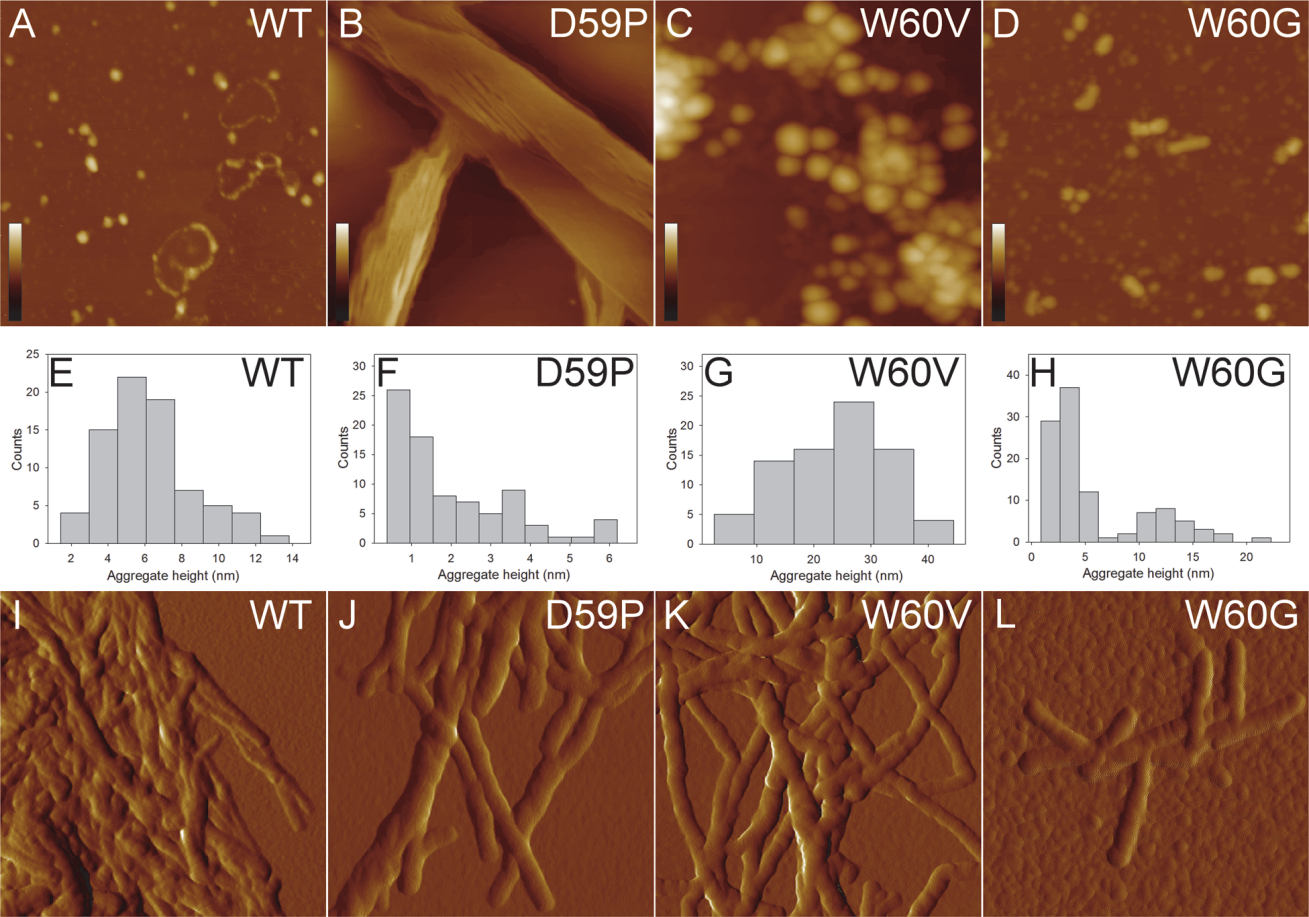
AFM characterisation of wt β2m and DE loop mutants aggregates incubated for 24 h. Tapping mode AFM images (top, height data; bottom, amplitude data) of wt β2m and DE loop mutants aggregated for 24h. A-D) Scan size 1.4 μm; the colour bars correspond to a Z range of A) 30 nm; B) 35 nm; C) 100 nm; D) 60 nm. E-H) histograms showing aggregate height measured from cross-sectional profiles in the topographic AFM images. I-L) fibrils found in the pellets of samples A-D). Scan size 860 nm.

Tapping mode AFM allowed us to inspect the morphologies of the β2m aggregates formed by the different variants. Representative images of the samples aggregated for 24 h, and deposited on the mica substrate after dilution, are reported in Fig. 2. Wt β2m mainly displayed isolated oligomers with a mean height of 6.2±0.3 nm, or oligomer chains (Fig. 2A and 2E), while the D59P variant extensively formed overlying planar sheets of filaments and thin fibrils, respectively about 1 and 4 nm high (Fig. 2B and 2F). The height of these fibrils is significantly lower than that measured for mature fibrils, suggesting that the thin fibrils are either intermediate structures or structures resulting from an epitaxial growth induced by the mica substrate. In any case, they reflect a strong tendency to fast aggregation, which is distinctive for this variant. Large spheroidal aggregates (mean height 25 ± 1 nm), probably resulting from oligomer clustering, were observed for the W60V variant (Fig. 2C and 2G). For the W60G variant, small oligomers (mean height 3.1 ± 0.2 nm) were found to coexist with larger oligomers (mean height 12 ± 1 nm), and very short protofibrils formed by few units of the latter (Fig. 2D and 2H). Mature fibrils were not observed for any of the variants, probably due to the relatively low fraction of fibrillar material. Therefore, samples were centrifuged and the pellet was analysed to check for the presence of mature fibrils. In all cases except for W60G β2m, mature fibrils were abundant in the pellet and were intertwined in clusters (Fig. 2I-K). A different behaviour was found for the W60G variant, which exhibited only very short, almost isolated, fibrillar structures (Fig. 2L).

**Fig 3:**
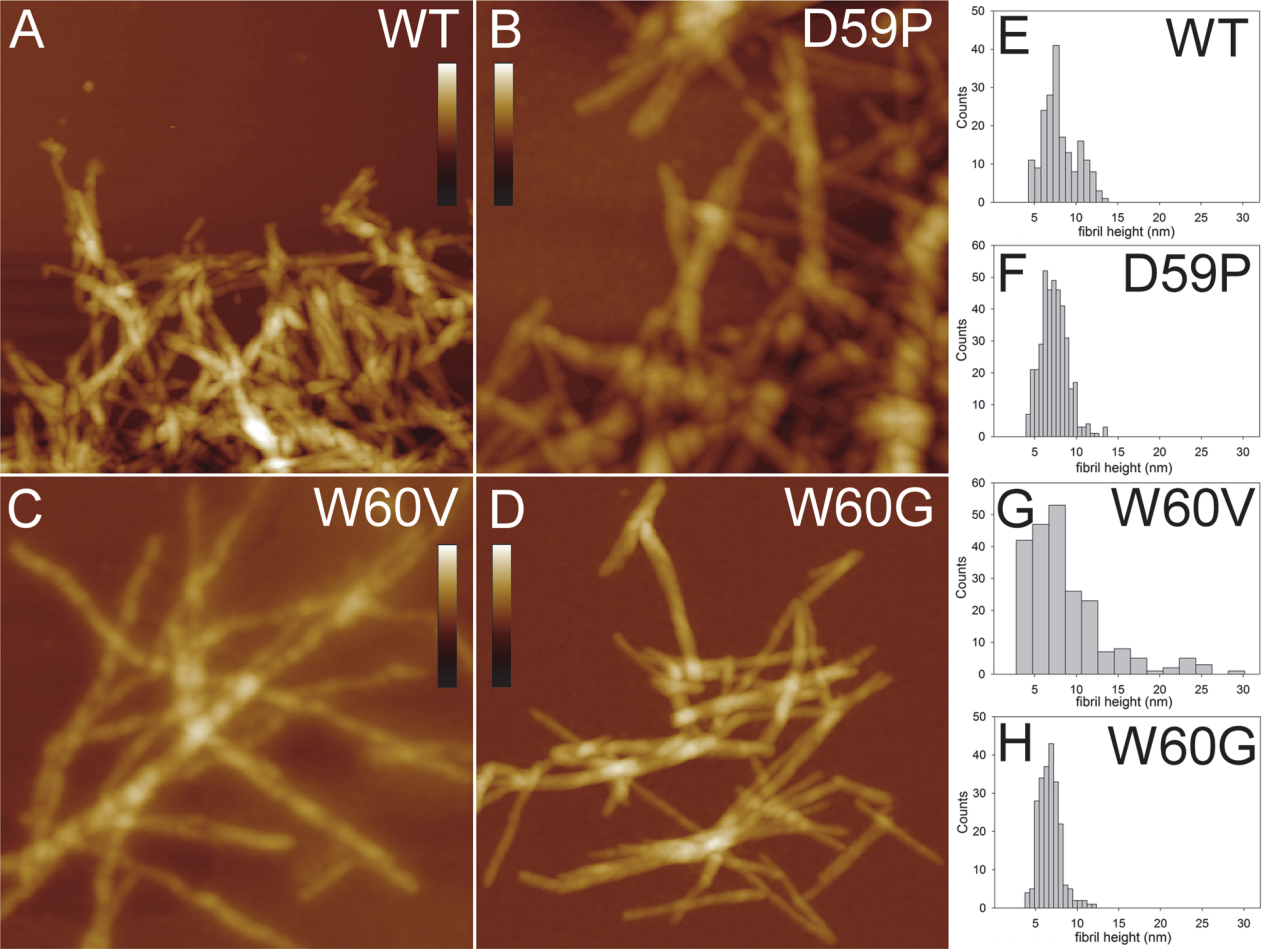
AFM characterisation of wt β2m and DE loop mutants aggregates incubated for one week. Tapping mode AFM images (height data) of mature fibrils of wt β2m and DE loop mutants obtained after one week incubation. Scan size 1.2 μm; the scale bars correspond to a Z range of: A and D) 55 nm; B) 70 nm; C) 65 nm. E-H) histograms of fibril height measured from fibril cross-sectional profiles in the topographic AFM images.

To increase the fraction of fibrillar material and better analyse mature fibrils from each protein variant, aggregated samples incubated for one week were prepared. Topographic images of the mature fibrils obtained for the wt and the different mutants after prolonged incubation are shown in Fig. 3A-D. Both wt β2m and the variants formed fibril clusters, but for the W60G mutant the cluster size was smaller than for the other variants. In many cases fibrils exhibit a twist with a periodicity varying between 40 and 60 nm. The fibril height distributions are reported in Fig. 3E-H. All the height distributions share a main peak at about 7 nm, indicating the presence of a common fibrillar structure as a main component. However, for W60V β2m the measured height values span a much broader range than in other cases, indicating the formation of more complex assemblies, which can still be recognized as isolated fibrils. W60G β2m fibrils are shorter than those formed by the other variants. The fibril mean lengths were (28±5)·10 nm, (70±1)·10 nm and (40±6)·10 nm for W60G β2m, W60V β2m and D59P β2m, respectively. For wt β2m, most fibrils were so closely intertwined that single fibrils could not be distinguished, preventing an estimate of the fibril length.

Collectively, the results of the AFM analysis suggest that a common mechanism of fibril formation takes place for wt β2m and the variants, although the details of the aggregation process can be different. The differences observed between the variants may be ascribed to their different aggregation propensities.

### Structure and dynamics of amyloid fibrils monitored by FTIR

**Fig 4:**
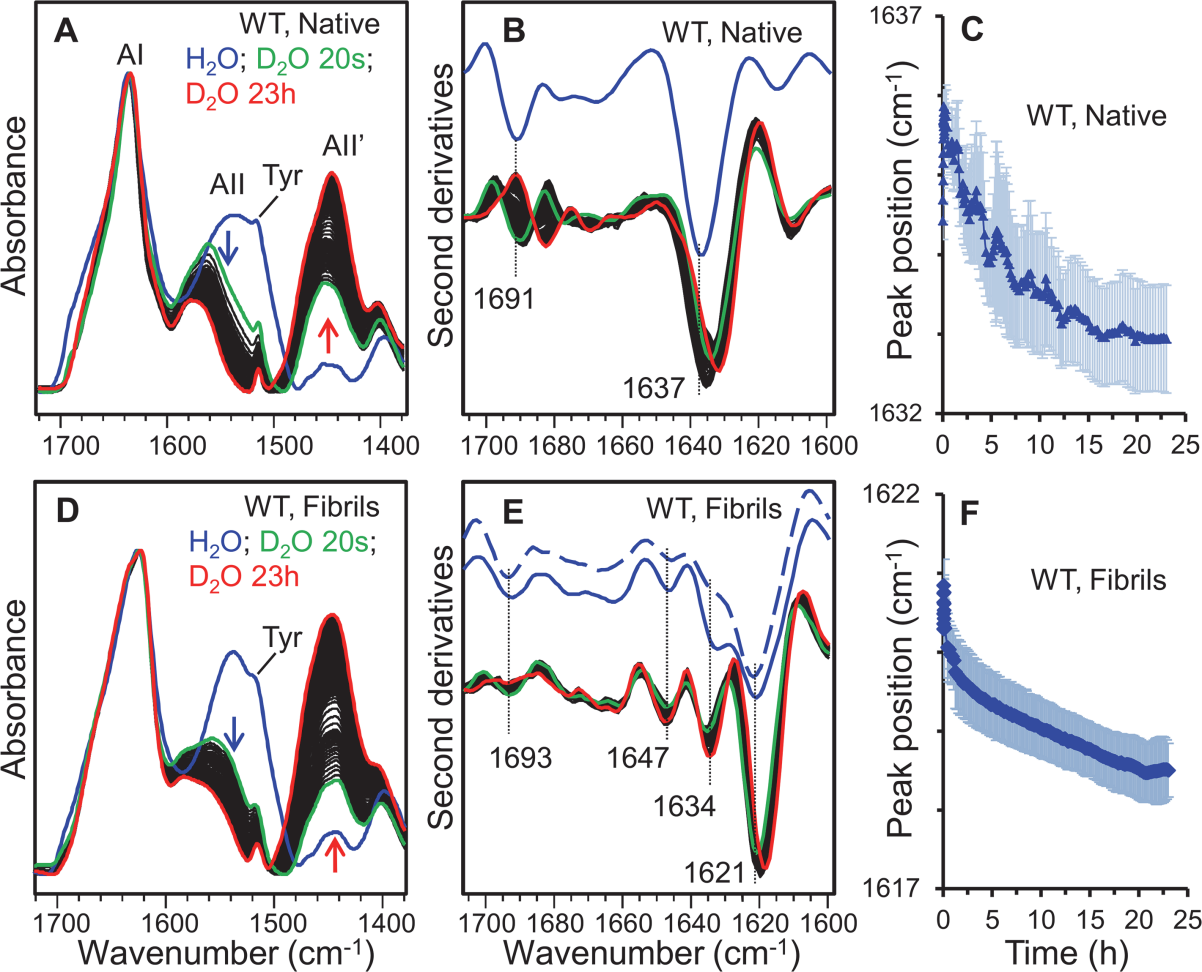
ATR/FTIR characterisation of wt β2m in the native and the fibrillar state. A) The absorption spectra of the native β2m in form of a protein film were collected before and after incubation in D_2_O for different times. Spectra are reported in the regions of Amide I (AI), Amide II (AII), and Amide II’ (AII’). Arrows point at increasing incubation time in D_2_O. Absorption spectra are normalized at the Amide I maximum. B) Second derivatives of the absorption spectra of (A) in the Amide I region. The spectra collected after D_2_O addition were normalized at the tyrosine band [27]. The marked peak positions of the two components due to the native antiparallel β-sheet structures refer to the spectrum of the undeuterated sample. C) Time course of the peak position of the main native β-sheet component reported after D_2_O addition to the protein film. Error bars represent the standard deviation of three independent samples. The peak positions were taken from the second derivative spectra. D) Absorption spectra of the wt β2m fibrils collected before and after incubation in D_2_O, reported as in (A). E) Second derivatives of the absorption spectra of (D) in the Amide I region. Spectra of two undeuterated fibrils obtained from independent preparations are compared to show fibril heterogeneity. The spectra collected after D_2_O addition were normalized at the tyrosine band [27]. The peak positions of the main components are indicated. F) Time course of the peak position of the main intermolecular β-sheet component is reported after D_2_O addition. Error bars represent the standard deviation of three independent fibril preparations. The peak positions were taken from the second derivative spectra.

The structural properties of the wt and DE loop β2m variants were examined by FTIR spectroscopy. In particular, we studied the fibril secondary structures and the hydrogen/deuterium (H/D) exchange kinetics of their core intermolecular β-sheet structures. As control experiments, the FTIR absorption spectra of the native wt β2m were also measured before and after D_2_O addition (Fig. 4A-C). To disclose the protein secondary structures the second derivatives of the absorption spectra were performed (Fig. 4B) [20, 21]. The Amide I band (1700–1600 cm^-1^) of native wt β2m is characterised by the two components at ~1637 cm^-1^ and at ~1691 cm^-1^ that can be assigned to the intramolecular antiparallel β-sheet structures of the protein, in agreement with previous FTIR characterisations [13, 22, 23]. Other minor components (Fig. 4B) around 1678 cm^-1^ and 1668 cm^-1^ (assigned to turns) and around 1614 cm^-1^ (likely due to β-sheets or amino acid side-chains) were observed [13, 22–24]. Upon the addition of D_2_O, a fast reduction of the band at 1535–1537 cm^-1^ (Amide II band mainly due to the amide groups NH bending vibrations) was found with a simultaneous increase of the band at 1443–1447 cm^-1^ (called Amide II’). These spectral changes can be taken as evidence of the H/D exchange (Fig. 4A) that can occur when the amide protons are replaced by deuterium in the accessible protein regions [25]. Actually, the H/D exchange is not only determined by the burial of the residues but also by the strength of the hydrogen bonds. Indeed, H/D exchange is reduced in secondary structures stabilized by strong hydrogen bonds, accordingly to the structure stability [25]. The exchange of the different protein secondary structures can be monitored by the downshift of their peak positions in the second derivative spectra, as reported in Fig. 4C. In particular, the main intramolecular β-sheet component of native wt protein downshifted from ~1637 cm^-1^ to ~1633 cm^-1^ (Fig. 4B and 4C), whereas the high-wavenumber β-sheet component downshifted from ~1691 cm^-1^ to ~1682 cm^-1^. The final peak positions of the native β-sheet components obtained in the D_2_O-hydrated film are in agreement with those found for the deuterated β2m in previous FTIR studies performed in transmission mode [13, 22, 26].

The FTIR absorption spectra of fibrillar wt β2m and their second derivatives are reported in Fig. 4D and 4E, respectively. The main Amide I component occurs around 1621 cm^-1^ that, with the 1693 cm^-1^ weaker component, can be unambiguously assigned to the intermolecular β-sheet structures of the undeuterated fibrils. The assignment of the additional components in the 1675–1634 cm^-1^ spectral region is not unequivocal. Indeed, as discussed in the literature [20, 22, 26–28], they can be assigned to turns (typically in the 1686–1660 cm^-1^ range), to loops (typically in the 1650–1640 cm^-1^ range), to native-like structures (around 1634 cm^-1^) or to a peculiar arrangement of the β-strands in the protein supramolecular assemblies [26]. We should note that the relative intensities of these components displayed a certain heterogeneity as observed for independent fibril preparations and illustrated in Fig. 4E, where two representative second derivative spectra of wt (undeuterated) fibril films are reported.

**Fig 5:**
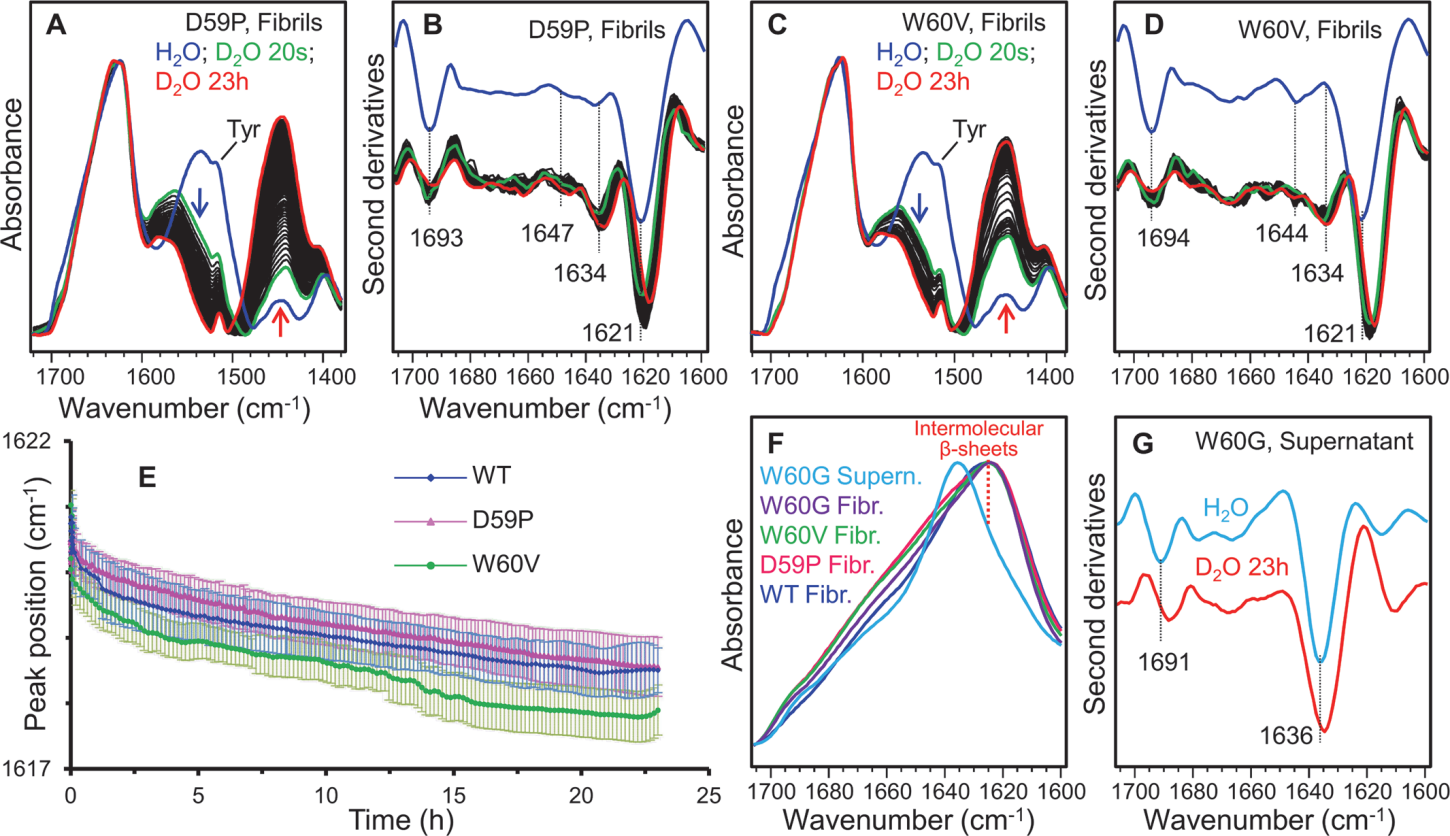
ATR/FTIR characterisation of DE loop mutants in the fibrillar state. A) The absorption spectra of the D59P fibrils were collected before and after incubation in D_2_O for different times. Spectra are reported in the regions of Amide I, Amide II, and Amide II’ bands. Arrows point to the spectral changes at increased incubation time in D_2_O. Absorption spectra are normalized at the Amide I maximum. B) Second derivatives of the absorption spectra of (A) in the Amide I region. The spectra collected after D_2_O additions were normalized at the tyrosine band [27]. The peak positions of the main components are indicated. C) The absorption spectra of the W60V fibrils were collected before and after incubation in D_2_O for different times and reported as in (A). D) Second derivatives of the absorption spectra of (C) in the Amide I region. E) Time course of the peak positions of the main intermolecular β-sheet component of wt, D59P, and W60V amyloid fibrils are reported after D_2_O addition to the protein films. Error bars represent the standard deviation of at least three independent fibril preparations. The peak positions were taken from the second derivative spectra. F) The absorption spectra of W60G, wt, D59P, and W60V fibrils and that of W60G supernatant are reported in the Amide I region. The intermolecular β-sheet structure absorption band is marked. G) Second derivative spectra of the W60G supernatant collected before and after 23 hours from D_2_O addition. The peak positions of the main components are indicated.

To study the solvent accessibility and dynamics [25] of the fibril core β-sheet structure, H/D exchange experiments were performed, as reported in Fig. 4D-F. In particular, during incubation in D_2_O, the main intermolecular β-sheet peak downshifted from ~1621 cm^-1^ in the undeuterated fibrils to ~1618 cm^-1^ after 23 hours of incubation in D_2_O (Fig. 4E and 4F). The same characterisations were performed on the fibrils of the DE-loop mutants (Fig. 5).

The undeuterated fibrils of D59P and of W60V variants share with aggregates of wt β2m the same two main Amide I components around 1693 cm^-1^ and 1621cm^-1^ that can be assigned to the intermolecular β-sheet structures (Fig. 5B and 5D). This result is in agreement with previous FTIR characterizations [13] on the same β2m variants studied here. Indeed, in these earlier experiments, the aggregation of unseeded wt and DE loop mutants took place inside the infrared cell, leading to final aggregates characterized by the same intermolecular β-sheet Amide I’ components.

Concerning the H/D exchange experiments presented in this work, as observed for wt fibrils, the main intermolecular β-sheet peak of D59P and of W60V fibrils was found to downshift from ~1621 cm^-1^ to around 1618 cm^-1^ after 23 hours of incubation in D_2_O (Fig. 5A-D). The time dependence of this peak position is reported in Fig. 5E for the wt, D59P, and W60V variants. A similar H/D exchange kinetics was observed for these three variants as can be seen from the partially overlapped standard deviations of independent fibril preparations (Fig. 5E). Indeed, the fibril second derivative spectra of the variants displayed similar Amide I components, with the main intermolecular β-sheet band peaked at the same wavenumber and characterized by a comparable H/D exchange (Figs. 4 and 5). These results suggest that the fibrils of the three variants are characterized by comparable intermolecular β-sheet structures and dynamics. However, the relative intensity of the other weaker Amide I components (at ~1647–1644 cm^-1^ and ~1634 cm^-1^) was found to vary in the final fibrils of the variants, indicating that minor structural rearrangements occurred in the presence of the mutations.

In agreements with the low aggregation propensity of the W60G mutant [13, 16], a low amount of fibrils was obtained by centrifugation of this sample after one-week incubation under fibrillogenic conditions. The absorption spectrum of the W60G fibrils displayed the same Amide I maximum as observed for the other variants (Fig. 5F). Unfortunately, the low amount of the collected W60G fibrils did not allow to obtain high quality ATR/FTIR spectra suitable for second derivative analysis and H/D exchange experiments and a significant amount of the W60G protein was instead found in the supernatant. In particular, the spectrum of the supernatant (Fig. 5F and 5G) displayed two main components at ~1691 cm^-1^ and at ~1636 cm^-1^, which after 23 hours incubation in D_2_O downshifted, respectively, to ~1688 cm^-1^ and ~1634 cm^-1^. These data suggest that the protein species in the W60G supernatant are characterized by native-like secondary structures.

All this considering, the FTIR results indicate that the wt β2m and the three DE loop variants investigated here (D59P, W60V, W60G) formed, under the same aggregation conditions, amyloid fibrils characterized by a common intermolecular β-sheet structure, with comparable H/D exchanges, and by limited structural differences among the different mutants. The previously reported low aggregation propensity of the W60G mutant was also confirmed here [13, 16].

## Discussion

Amyloid aggregation is a life threatening process, which is at the basis of many severe pathologic conditions. Protein aggregation is a complex process, and the heterogeneity of such process adds serious challenges to its structural and biophysical characterisation. The lack of detailed structural data on amyloid formation undermines our understanding of the pathologic process and hampers the design of pharmacological therapies. Over the last 10–20 years one of the most successful strategies to gather biochemical and biophysical evidence on protein aggregation has been to mutate the polypeptide sequence, testing the aggregation properties of the mutated variants, thus inferring the role played by selected residues during amyloid aggregation. However, mutations may introduce several independent effects; mutations can subtly affect the structure of the protein, its dynamics, its thermodynamic stability, the energetic barriers between different states *etc*. Therefore, not only is it crucial to observe that a modified aggregation propensity stems from a specific mutation, but it is also necessary to understand which effect(s) the mutation exerts on aggregation end point. In particular, many cases are known where mutations lead to off-pathway states or trigger a different kind of aggregation. The elegant crystal structure of hexameric β2m reveals many interesting intermolecular interactions, however such hexameric form does not aggregate under standard conditions [29], leaving open the question on which are the interactions that set the hexamer off-pathway. Specific mutations may structurally protect the protein from the aggregation pathway undertaken by the wt protein, but in parallel open new paths to amyloid formation, as recently exemplified by protective mutations on Acylphosphatase from *Sulfolobus solfataricus* [30, 31].

To date, using different techniques, we have shown that many marked effects stemmed from mutations in the DE loop: thermodynamic stability and aggregation propensity vary according to the DE loop geometry [15, 18]. However, in order to draw conclusions on the role of the DE loop on β2m amyloid aggregation it is crucial to establish whether the final step of aggregation (*i*.*e*. the amyloid fibrils) is maintained. Should the starting and the end points of the aggregation process be conserved in wt β2m and in the mutants, we could propose a conservation of the aggregation process for the four explored variants. In such a scenario the effects previously observed upon mutations in the DE loop could be discussed in the context of the wt protein aggregation process. Conversely, formation of aggregates by the DE loop mutants not sharing structural features with those displayed by the wt protein would imply the onset of distinct aggregation pathway(s), ultimately preventing us from assigning any role to the DE loop in the aggregation of wt β2m. Based on such considerations, we deemed relevant to investigate the nature of the amyloid aggregates produced by the DE loop mutants, compared to those formed by wt β2m, to clarify the role played by the loop on β2m amyloid propensity.

First, quantitative information about aggregate size and shape has been obtained by AFM. The images acquired from samples incubated for 24 hours confirm, as previously reported [13], that wt β2m and the three DE loop variants display different aggregation kinetics. In particular, D59P β2m exhibited a fast aggregation, giving rise to sheets of thin fibrils, which were not observed either in wt β2m or in the other variants. W60G β2m showed the slowest aggregation, as after 24 hours fibrils were rare and short even in the pellet, differently from the other samples. In the pellets of samples incubated for a week, mature fibrils were found for wt β2m and the three variants. The analysis of fibrils cross-sectional profiles indicates that in all cases the fibril populations share a peak at about 7 nm in the height distributions. The fibril heights observed for the samples analysed in the present study are consistent with those reported by Ohhashi *et al*. for wt β2m fibrils formed at neutral pH by ultrasonication in the presence of SDS [32]. Although these aggregation conditions are not the same as in our study, they can be considered as somewhat equivalent; in fact, it has been observed that both TFE and SDS act as hydrophobic co-solvents favouring fibrillation [33].

The AFM analysis indicates that despite differences in the aggregation kinetics, the wt β2m and three mutants give rise to fibrils with comparable heights that suggest a common fibrillar architecture. Moreover, the secondary structure content detected by ATR/FTIR in mature aggregates, shows that all four kind of fibrils are characterised by the same Amide I components due to the intermolecular β-sheet structures of the final protein assemblies. Finally, H/D exchange was employed to evaluate structural dynamics, compactness, and stability, providing information on fibril molecular packing [25]. In keeping with the biophysical characterizations reported above, H/D exchange kinetics observed for wt β2m and the DE loop mutants aggregates are well comparable, further suggesting that the overall fibril assembly is shared among the four protein variants.

## Conclusions

In summary, the data presented here suggest that the mutations in the DE loop do not alter significantly the overall structural properties of the β2m amyloid aggregates. Given that β2m native fold and the mature fibrillar aggregates appear unaltered ([18] and this work), we propose that the aggregation process is conserved for the β2m mutants examined here, and that the DE loop is a crucial region determining the wt β2m aggregation propensity, affecting the aggregation kinetics. In particular, it has been found that the DE loop has a strong thermodynamic influence on the β2m native state. This loop is a source of instability that likely determines the energies associated with the different folded states and the energetic barriers between them, resulting in the aggregation propensity observed for monomeric wt β2m. By simple modifications of residues and of the geometry in this loop it is possible to tune the stability of the β2m fold and to practically abolish (or to increase) β2m amyloid formation. Such observations can be reconciled with the evolution of β2m as structural part of the MHC-I complex. Notably the DE loop of human β2m has the main role of properly orienting Phe56 (just upstream the loop) and Trp60 for the interaction with the heavy chain in the MHC-I (Fig. 1B). The presence and the positioning of the bulky Trp60 side chain is ideal for the interaction with an amphipathic cleft of the neighbouring heavy chain; therefore in general the loop strained geometry and the overall β2m fold are efficiently stabilised by the tight interactions between β2m and the heavy chain in the MHC-I complex [17]. However, once wt β2m is released in the blood as a monomer, even if it is globally very stable, it presents in the DE loop all the ingredients for misfolding: a conformational strain which makes the D strand and DE loop region flexible and unstable [16], and a patch of solvent-exposed aromatic side chains in the D and E strands and in the DE loop, which will drive an overall entropy gain upon protein aggregation (see also [34]).

## Methods

### Sample preparation

Wt β2m and the three mutated variants (W60G, D59P, W60V) were expressed and purified as previously described [16].

The aggregated samples were prepared as follows: 100 M β2m was incubated at 37°C under shaking (Mixing-Block MB102, BIOER orbital shaking with 3mm amplitude at 600 rpm) in 50 mM Na phosphate buffer, 100 mM NaCl, pH 7.4, in the presence of 20% (v/v) TFE [35]; 20 μg/ml of β2m fibril seeds were added to the samples. Wt β2m and its variants were incubated for 24 h or for one week under the aggregation conditions. The formation of fibrillar aggregate has been monitored by measuring Thioflavin T (ThT) fluorescence according to LeVine [36].

### Atomic force microscopy

For AFM inspection, samples were diluted 500-fold and a 10 μl aliquot was deposited on a freshly cleaved mica substrate, and dried under mild vacuum. Alternatively, to recover fibrillar material, samples were centrifuged at 1700 x g for 10 min using an Eppendorf 5417R centrifuge, the pellet was suspended in an equal volume of water, and a 10 μl aliquot was deposited on mica and dried under mild vacuum.

AFM images were acquired in tapping mode in air using a Dimension 3100 Scanning Probe Microscope equipped with a ‘G’ scanning head (maximum scan size 100 μm) and driven by a Nanoscope IIIa controller, and a Multimode Scanning Probe Microscope equipped with “E” scanning head (maximum scan size 10 μm), driven by a Nanoscope IV controller (Digital Instruments—Bruker). Single beam uncoated silicon cantilevers (type OMCL-AC160TS, Olympus) were used. The drive frequency varied between 270 and 330 kHz, the scan rate was between 0.5 and 0.8 Hz. Aggregate size was measured from the corresponding height profiles obtained from topographic AFM images.

### Fourier transform infrared spectroscopy

The infrared absorption spectra were collected in the attenuated total reflection (ATR) mode on a single reflection diamond element (Golden Gate, Specac, USA). The fibrils of the β2m variants, obtained by centrifugation at 17000 x g for 15 min at 4°C and resuspended in the fibrillogenesis buffer for a second centrifugation, were transferred to the ATR plate and dried at room temperature in order to obtain a protein hydrated film [25, 37, 38]. In order to study the hydrogen/deuterium (H/D) exchange of the sample, the FTIR spectra were collected on the protein films before and after the addition of 3μL of D_2_O [25]. During the analyses, the sample on the ATR plate was covered in order to avoid solvent evaporation. FTIR measurements were performed using the Varian 670-IR spectrometer (Varian Australia Pty Ltd, Mulgrave VIC, Australia) under the following conditions: 2 cm^-1^ resolution, a scan speed of 25 kHz, triangular apodization, and a nitrogen-cooled Mercury Cadmium Telluride detector. In order to follow the H/D exchange kinetics, the number of scan coadditions was adjusted from 15 (immediately after D_2_O addition) to 2000 (starting from about 6 hours after D_2_O addition). The measured spectra were smoothed by the Savitsky-Golay method before the second derivative analysis, both performed by the Resolutions-Pro software (Varian Australia Pty Ltd, Mulgrave VIC, Australia). As controls, the same FTIR characterisations have been performed on the native wt β2m (at 400 μM concentration in Na phosphate buffer 50mM, pH 7.4) and on the supernatant of W60G mutant. In this last case, after one week incubation under fibrillogenesis conditions, the W60G sample was centrifuged at 17000 x g for 15 min at 4°C and the supernatant was concentrated three times by a centrifugal filter device (Microcon, Millipore Corporation, Bedford, MA, USA) at 4°C. The protein film, obtained from a supernatant volume of 3μL, was subjected to the FTIR characterisations as described above for the β2m fibrils.
